# Contralateral seventh cervical nerve transfer can affect the pennation angle of the lower limb in spastic hemiplegia patients: An observational case series study

**DOI:** 10.1002/brb3.1460

**Published:** 2019-11-13

**Authors:** Bao‐Fu Yu, Li‐Wen Chen, Yan‐Qun Qiu, Jing Xu, Hua‐Wei Yin, Qin‐Ying Li, Wen‐Dong Xu

**Affiliations:** ^1^ Department of Hand Surgery Huashan Hospital Fudan University Shanghai China; ^2^ Department of Hand and Upper Extremity Surgery Jing'an District Center Hospital Shanghai China; ^3^ Shanghai Clinical Medical Center for Limb Function Reconstruction Shanghai China; ^4^ Shanghai Key Laboratory of Peripheral Nerve and Microsurgery Shanghai China; ^5^ National Clinical Research Center for Aging and Medicine Huashan Hospital Fudan University Shanghai China

**Keywords:** cervical nerve transfer, interlimb interaction, load distribution, pennation angle, spastic hemiplegia, ultrasonography

## Abstract

**Introduction:**

We previously reported transferring seventh cervical (C7) nerve from unaffected side to affected side in patients with spastic hemiplegia due to chronic cerebral injury, to improve function and reduce spasticity of paralyzed upper limb. In the clinics, some patients also reported changes of spasticity in their lower limb, which could not be detected by routine physical examinations. Pennation angle of muscle can indirectly reflect the condition of spasticity. The purpose of this study was to evaluate whether this upper limb procedure may affect spasticity of lower limb, using ultrasonography to detect changes of muscle pennation angle (PA).

**Methods:**

Twelve spastic hemiplegia patients due to cerebral injury including stroke, cerebral palsy, and traumatic brain injury, who underwent C7 nerve transfer procedure, participated in this study. B‐mode ultrasonography was used to measure PA of the gastrocnemius medialis (GM) muscle at rest preoperatively and postoperatively. The plantar load distribution of the lower limbs was evaluated using a Zebris FDM platform preoperatively and postoperatively.

**Results:**

The PA of the GM was significantly smaller on the affected side than that of unaffected side before surgery. On the affected side, the postoperative PA was significantly larger than preoperative PA. On the unaffected side, the postoperative PA was not significantly different compared to preoperative PA. The postoperative plantar load distribution of the affected forefoot was significantly smaller than preoperative load distribution, which was consistent with ultrasonography results.

**Conclusions:**

This study indicates that C7 nerve transfer surgery for improving upper limb function can also affect muscle properties of lower limb in spastic hemiplegia patients, which reveals a link between the upper and lower limbs. The interlimb interactions should be considered in rehabilitation physiotherapy, and the regular pattern and mechanism need to be further studied.

## Graphical abstract

This study indicates that seventh cervical nerve transfer for upper limb can also affect muscle properties of lower limb in spastic hemiplegia patients, revealing a link between the upper and lower limbs, which should be considered in rehabilitation physiotherapy.

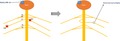

## 1 INTRODUCTION

For patients with spastic hemiplegia due to central nervous injury, we previously reported transferring contralateral seventh cervical (C7) nerve from unaffected side to affected side to restore the function of affected upper limb (Zheng et al., 2018). This procedure has been verified to be able to reduce spasticity and improve the function of affected upper limb. It has not been studied whether this procedure can affect the function of the lower limb. During clinical follow‐up of spastic hemiplegia patients who underwent contralateral C7 nerve transfer, we observed that some patients walked differently compared with preoperative conditions. Some patients also reported changes of spasticity in their lower limb after this surgery. We hypothesized that contralateral C7 nerve transfer for spastic upper limb paralysis can also have effects on the function of the lower limb in spastic hemiplegia patients.

For spastic hemiplegia patients, one of the main factors resulting in dysfunction of upper and lower limbs is high muscle tone (Pradines et al., 2018). Although the Modified Ashworth Scale (MAS) and the Modified Tardieu Scale (MTS) are quick and easy to perform in clinical evaluation of spasticity, they are subjective measurements and not relatively sensitive to detect unobvious changes of spasticity (Aloraini, Gaverth, Yeung, & MacKay‐Lyons, 2015; Burridge et al., 2005). The geometric arrangement of muscle fibers in skeletal muscle determines the function of muscle. Therefore, measuring the morphological changes of muscle fibers can be an alternative assessment of muscle activity (Lieber & Friden., 2000). The Pennation angle (PA) is one of the most commonly used anatomic parameters of skeletal muscle, reflecting the function condition of muscle (Zajac, 1992). A larger PA means less tension relative to the corresponding tendon (Li, Tong, & Hu, 2007; Zajac, 1992). Therefore, measuring changes of PA can indirectly assess spasticity of limbs.

Ultrasonography has been a commonly used method to character muscle architecture, since it is very safe and noninvasive (Narici, 1999). In vivo, ultrasonography imaging (USI) is usually used for assessment of skeletal muscles. Previous studies have shown good intra‐ and interexaminer reliability of the gastrocnemius medialis (GM) muscle PA evaluated with USI in poststroke patients, and the PA measured by USI was clinically useful assessment of rehabilitation effects in poststroke patients (Cho, Cho, Yoo, Lee, & Lee, 2018; Cho, Lee, & Lee, 2014). Spastic hemiplegia patients usually present with foot‐drop and their ankle dorsiflexion was impaired, which is associated with the increased muscle tone of the calf muscles (Berenpas et al., 2018; Singer, Dunne, & Allison, 2001; Soltani, Rahimi, Naimi, Khademi, & Saeedi, 2014). Spastic foot‐drop would also increase plantar load distribution of the forefoot on the affected side (Berenpas et al., 2018; Soltani et al., 2014). The lateral gastrocnemius, GM, and the soleus consist of the triceps surae muscle group, and determine the plantar flexors of the ankle. Therefore, we chose the GM as a representative muscle of the calf muscles, to detect the effects of contralateral C7 nerve transfer surgery on lower limbs in spastic hemiplegia patients.

In this study, we used ultrasonography to measure the PA of the GM preoperatively and postoperatively. We hypothesized that contralateral C7 nerve transfer surgery for improving the function of upper limb can change the PA of the GM muscle in spastic hemiplegia patients. As a result of the changes of muscle properties, we hypothesized that the load distribution of the lower limbs would also change after the surgery.

## 2 MATERIALS AND METHODS

### 2.1 Participants

A total of 12 spastic hemiplegia patients due to cerebral injury including stroke, cerebral palsy, and traumatic brain injury, who hospitalized in our medical center for contralateral C7 nerve transfer procedure, were selected to participate in this study. They primarily wanted to improve the function of their spastic upper limb through this surgery. The MAS score was measured at the ankle. The selection criteria for the patients included the followings: (a) spastic hemiplegia resulting from cerebral injury for more than 3 years; (b) the MAS score of the affected ankle was larger than 1 (maximal value, 4); (c) no surgical procedure performed on the affected lower limbs; (d) no complications during perioperative period. Exclusion criteria were as follows: (a) history of surgery on the affected lower limbs; (b) no obvious spasticity on the lower limbs; (c) refusing to be followed up regularly. This study was approved by the Institutional Review Board of Jing'an District Center Hospital and was conducted according to the ethical guidelines of the Helsinki Declaration. All patients gave informed consent before participating in the study.

### 2.2 Clinical evaluation

Medical history of patients recruited into the study was recorded mainly including cause of brain injury, years postinjury, paralyzed side before the contralateral C7 nerve transfer surgery. The MAS was used to evaluate the conditions of spasticity of the ankle dorsiflexion. The MAS is a measure of muscle tone (spasticity), and its scores range from 0 to 4 at each joint (Aloraini et al., 2015; Burridge et al., 2005). Higher scores of the MAS indicate severer spasticity (Aloraini et al., 2015; Burridge et al., 2005).

### 2.3 Contralateral C7 nerve transfer surgery

**Figure 1:**
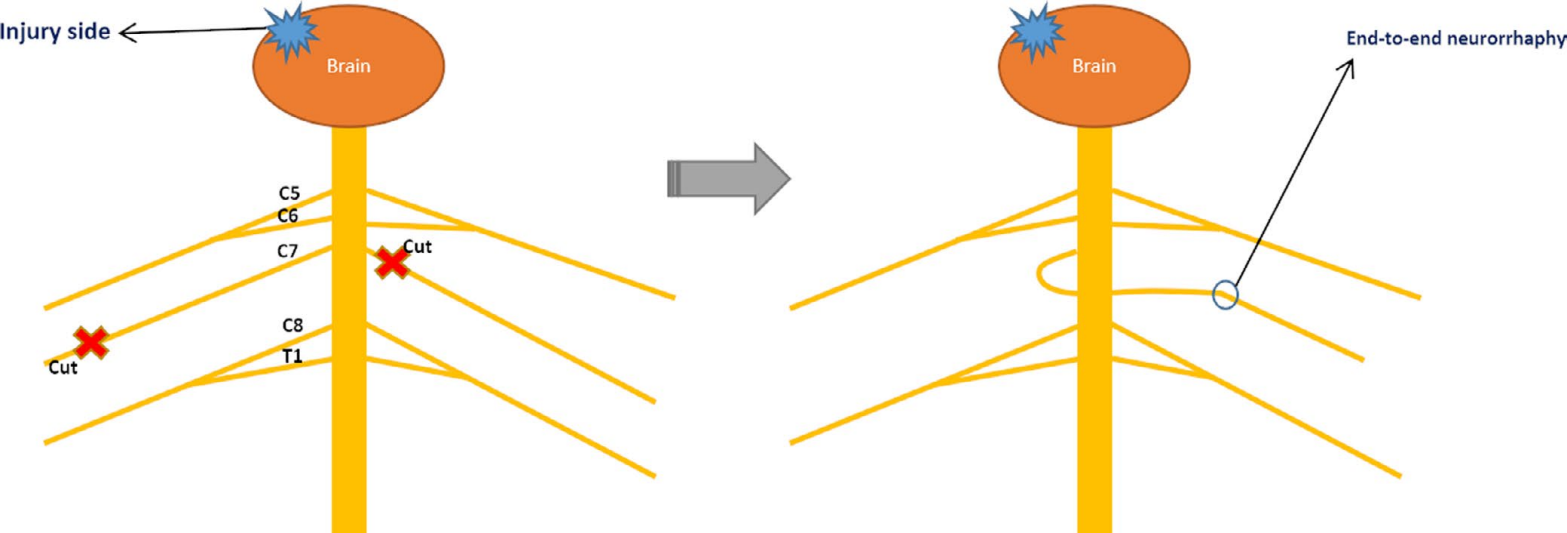
The diagrammatic sketch of contralateral seventh cervical (C7) nerve transfer surgery. The C7 nerve on the paralyzed side is cur near the intervertebral foramen, and the C7 nerve on the nonparalyzed side is cut as distally as possible, proximal to the point at which it combines with the fibers of other brachial plexus nerves. The cut end of the C7 nerve on the nonparalyzed side was drawn through the prespinal route to the paralyzed side and anastomosed directly (without a graft) to the cut end of the C7 nerve on the paralyzed side by means of microsurgical epineurium suturing (Zheng et al., 2018)

We have described this surgery in detail in previous study (Zheng et al., 2018). In simple terms, an approximately 15‐cm transverse incision was performed at the bottom of the neck. After bilateral brachial plexus nerves were exposed, the C7 on the affected side was cut near the foramen intervertebrale, and the contralateral C7 was cut near the point where other brachial plexus nerves combine with the C7. End‐to‐end neurorrhaphy was performed between the cut end of affected side and the cut end of unaffected side through the prespinal route with microsurgical suture. The diagrammatic sketch of contralateral C7 nerve transfer surgery is shown in Figure 1. After this procedure, a head–arm brace was used to immobilize the paralyzed upper limb for 4 weeks. Then, the patients underwent regular rehabilitation therapy for the affected upper limb, which mainly included identical active exercise, passive range of motion, occupational therapy, functional training, physical therapy, acupuncture, and massage (Zheng et al., 2018). Rehabilitation therapy for lower limb was not performed.

### 2.4 Measuring the PA with ultrasonography

**Figure 2:**
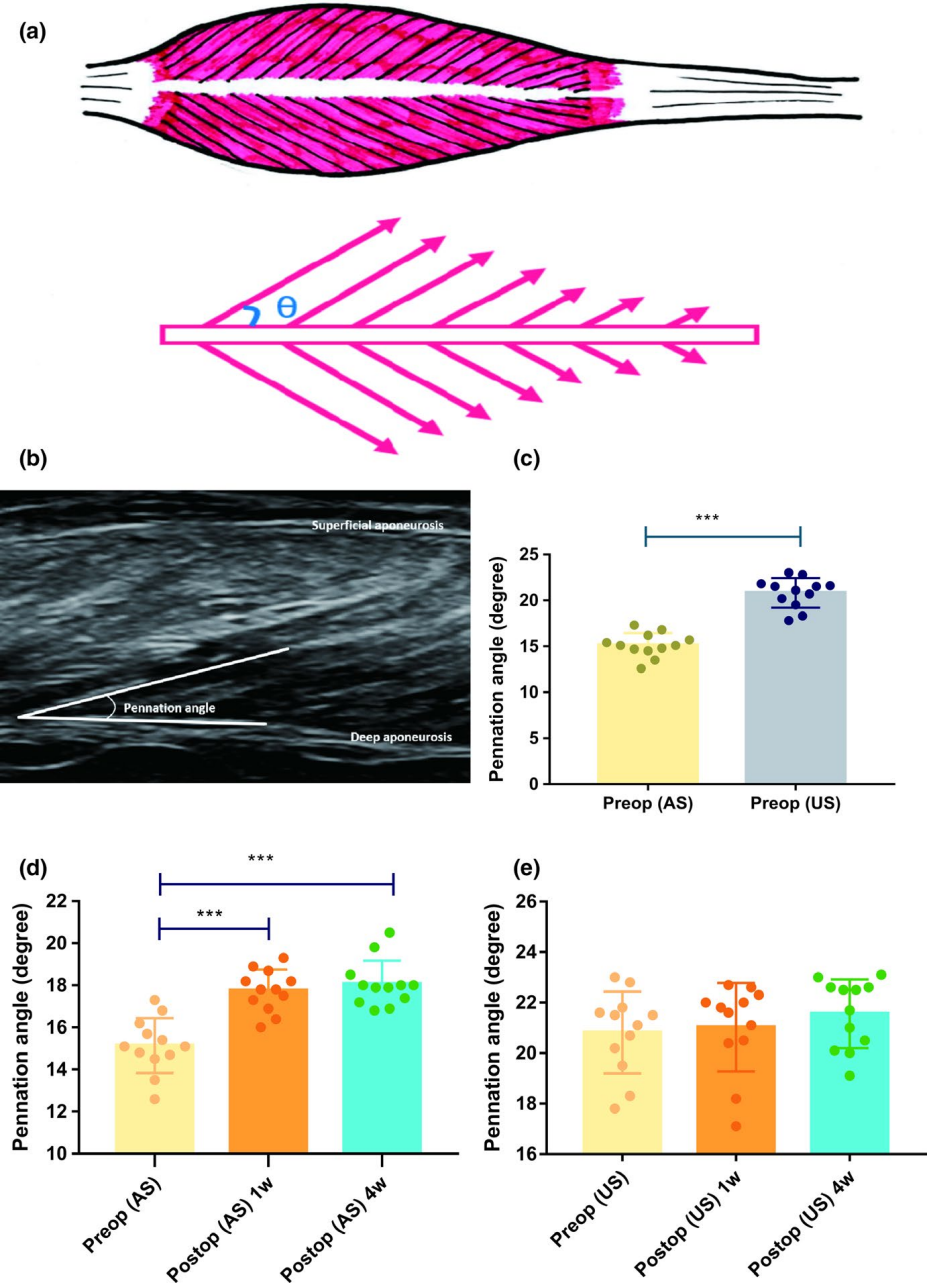
The pennation angle measured by ultrasonography. (a) The diagrammatic sketch of geometric arrangement of muscle fibers in pennate muscle. Pennation angle (*θ*) was defined as the angle of insertion of the muscle fascicle into the deep aponeurosis. (b) Pennation angle is shown on ultrasonography imaging. Comparisons of the pennation angle measured by ultrasonography. (c) Comparison of preoperative pennation angle between affected and unaffected lower limbs. (d) Comparison of pennation angle between preoperative and postoperative values on the affected lower limb. (e) Comparison of pennation angle between preoperative and postoperative values on the unaffected lower limb. AS, affected side; Postop, postoperation; Preop, preoperation; US, unaffected side. ***p < .001

In this study, we used B‐mode ultrasonography to measure the PA of the GM muscle. Using a 14‐MHz high‐resolution probe, an experimenter with 8 years of experience in ultrasonic measurements collected ultrasonic images of the GM muscle by placing the probe at 30% proximal to the medial malleolus of the fibula and the medial condyle of the tibia, according to literature (Cho et al., 2018; Mathevon et al., 2018). The PA was defined as the angle of insertion of the muscle fascicle into the deep aponeurosis (Aggeloussis, Giannakou, Albracht, & Arampatzis, 2010; Kwah, Pinto, Diong, & Herbert, 2013; Zajac, 1992; Figure 2a,b). During the measuring period, the patients were in a prone position with knee extension posture, and the GM muscle was relaxed. Three times scan wase performed, and the averaged values of these measurements were calculated and used for further data analysis. The PA of bilateral GM muscles was measured before the surgery (baseline), at postoperative 1 and 4 weeks by the same experimenter with the probe placed at the same position.

### 2.5 Evaluating plantar load distribution of the lower limbs

**Figure 3:**
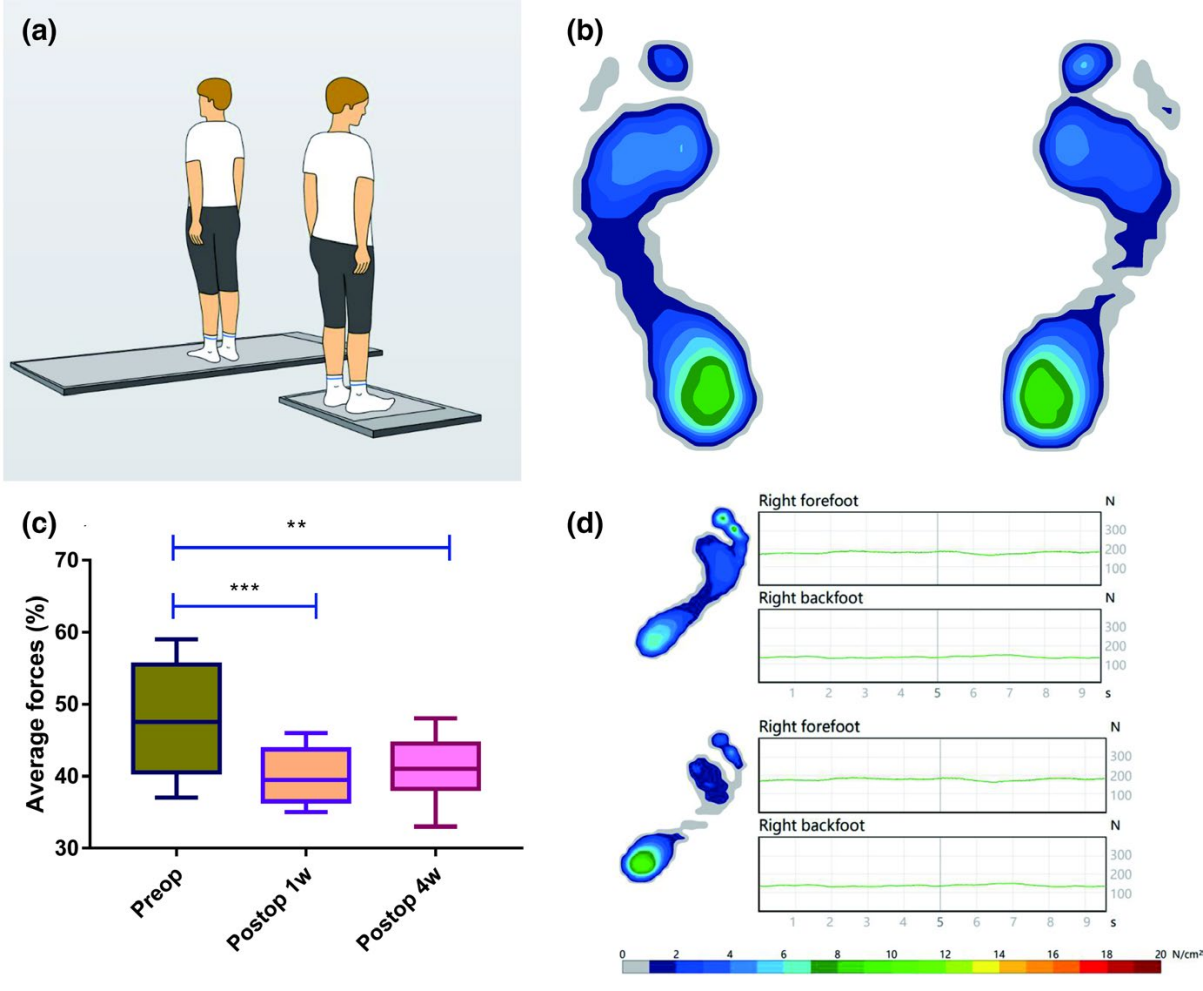
Plantar load distribution of the lower limbs. (a) The diagrammatic sketch from the homepage of Zebris FDM software shows the test was performed in a bipedal position. (b) In the Zebris FDM software, the pressure map of plantar load distribution of a healthy people was created. (c) Comparison of the plantar load distribution of affected forefoot between preoperative and postoperative values. (d) A typical case whose postoperative plantar load distribution of affected forefoot was significantly reduced compared to that of preoperative load with backfoot load increasing significantly. ***p < .001; **p < .01

A pedobarographic platform from Zebris^®^ Medical GmbH was used to evaluate plantar load distribution of the lower limbs (Kalron, Nitzani, & Achiron, 2016; Morasiewicz, Konieczny, et al., 2018; Morasiewicz, Urbański, et al., 2018). The platform was equipped with 1,504 sensors and connected to a PC. Patients were evaluated barefoot, with eyes open. Before test, the device was calibrated, and patients were taught the method of test. Patients were all familiar with specific posture before testing. The test was performed for 10 s in a bipedal position (Figure 3a). Each patient underwent the test three times and the average scores of these measurements were recorded for further analysis (Ko & Lee, 2013). The Zebris FDM software (https://www.zebris.de/en/) was used to process and record pressure parameters that were later subjected to statistical analysis (Kalron et al., 2016; Morasiewicz, Konieczny, et al., 2018; Morasiewicz, Urbański, et al., 2018). In the Zebris FDM software, the pressure map of plantar load distribution was created (Figure 3b; Ko & Lee, 2013; Morasiewicz, Konieczny, et al., 2018; Morasiewicz, Urbański, et al., 2018; Soltani et al., 2014)^19–22^. Load distribution of forefoot on the affected side was expressed as percentage of the load of the same foot. For spastic hemiplegia patients, their spastic foot‐drop would increase plantar load distribution of the forefoot on the affected side (Daniilidis et al., 2017; Tanıgör, Karabulut, Çelebisoy, Eraslan, & Zihni, 2019). When the spasticity reduces, the load distribution of the forefoot would reduce correspondingly, and the load distribution of the backfoot would increase, which is more similar to healthy control people (Daniilidis et al., 2017; Tanıgör et al., 2019).

### 2.6 Statistical analysis

Statistical analysis was carried out using the SPSS 19.0 software package (IBM Corp). Comparisons between preoperative PA on the affected side and unaffected side, preoperative and postoperative PA on the affected side, preoperative and postoperative PA on the unaffected side, preoperative and postoperative Plantar load distribution were conducted with Wilcoxon signed‐rank test. Statistical significance was defined as a two‐sided *p* value of <.05.

### 2.7 Ethical publication statement

We confirm that we have read the Journal's position on issues involved in ethical publication and affirm that this report is consistent with those guidelines.

## 3 RESULTS

### 3.1 Demographic and clinical data

All 12 patients were regularly followed up for more than 1 month, and no patients dropped out or lost to follow‐up. Clinical characteristics of the 12 patients before contralateral C7 nerve transfer procedure were shown in Table 1. These patients were between 17 and 65 years old (mean, 48.83 years). Nine patients were males (75%). Cause of brain injury included stroke (9/12), cerebral palsy (1/12), and traumatic brain injury (2/12). Affected side of eight patients was right (8/12). The MAS scores of the ankle dorsiflexion ranged from 1^+^/4 to 3/4.

**Table 1:** Clinical characteristics of the 12 patients before contralateral seventh cervical nerve transfer surgery

Patients	Age (year)	Sex	Cause of injury	Years Postinjury	Affected side	MAS score (lower limb)	MAS score (upper limb)
1	48	F	Stroke	5	L	2	2
2	55	M	Stroke	4	R	2	1
3	17	F	Cerebral palsy	17	R	2	2
4	62	M	Stroke	4	L	1+	2
5	56	M	Stroke	5	R	2	1+
6	49	M	Stroke	3	R	2	2
7	52	M	Stroke	5	R	2	1
8	27	F	Traumatic brain injury	5	L	3	2
9	65	M	Stroke	7	R	1+	2
10	45	M	Stroke	6	R	1+	2
11	59	M	Stroke	5	L	3	2
12	51	M	Traumatic brain injury	6	R	2	1+

Abbreviations: F, female; M, male; MAS, Modified Ashworth Scale.

### 3.2 The PA measured with ultrasonography

Before the surgery, the PA of GM muscle was 15.14 ± 1.31° (Mean ± *SD*) on the affected lower limb and 20.82 ± 1.62° on the unaffected side. The mean PA on the affected side was significantly smaller than that on the unaffected side (difference, −5.68; 95% confidence interval [CI], −7.3 to −4.02; *p* < .001; Figure 2c).

On the affected side, the PA at postoperative 1 week was 17.75 ± 1.00° and was significantly larger than that of preoperative value (difference, 2.61; 95% CI, 0.95–4.27; *p* < .001; Figure 2d). The PA on the affected side at postoperative 4 weeks was 18.08 ± 1.10° and was significantly larger than that of preoperative value (difference, 2.93; 95% CI, 1.28–4.59; *p* < .001; Figure 2d). The difference of mean PA between postoperative 1 and 4 weeks was not significant (difference, −0.33; 95% CI, −2.04 to 1.39; *p* > .05; Figure 2d).

On the unaffected side, the PA at postoperative 1 week was 21.03 ± 1.75° and was not significantly different compared to that of preoperative value (difference, 0.21; 95% CI, 1.45–1.87; *p* > .05; Figure 2e). The PA on the unaffected side at postoperative 4 weeks was 21.56 ± 1.36° and was not significantly different compared to that of preoperative value (difference, 0.74; 95% CI, 0.91–2.40; *p* > .05; Figure 2e). The difference of mean PA between postoperative 1 and 4 weeks on the affected side was not significant (difference, −0.53; 95% CI, −2.19 to 1.12; *p* > .05; Figure 2e).

### 3.3 Plantar load distribution of the lower limbs

Before the surgery, the plantar load distribution of affected forefoot was 48.17 ± 7.59%. The plantar load distribution of affected forefoot at postoperative 1 week was 40.08 ± 3.80% and was significantly smaller than that of preoperative value (difference, −8.08; 95% CI, −11.38 to −4.78; *p* < .001; Figure 3c). The plantar load distribution of affected forefoot at postoperative 4 weeks was 41.25 ± 4.12% and was significantly smaller than that of preoperative value (difference, −6.92; 95% CI, −10.81 to −3.02; *p* < .01; Figure 3c). The difference of plantar load distribution of affected forefoot between postoperative 1 and 4 weeks was not significantly (difference, 1.16; 95% CI, −0.10 to 2.43; *p* > .05; Figure 3c). Figure 3d shows a typical case whose postoperative plantar load distribution of affected forefoot was significantly reduced compared to that of preoperative load.

## 4 DISCUSSION

We previously reported transferring contralateral C7 nerve from unaffected side to affected side in treating spastic hemiplegia due to chronic cerebral injury. The clinical effects of this surgery were tested, and it was demonstrated that this surgery can significantly produce more improvements of the function of the paralyzed upper limb than physical therapy alone (Zheng et al., 2018). Recently, we also reported the contralateral lumbar to sacral nerve transfer for the lower limbs in hemiplegic patients after stroke, making significant improvements on the function of the affected lower limb (Qiu et al., 2019). However, this interesting phenomenon has not been reported previously that peripheral nerve transfer surgery for upper limb can also have effects on the function of lower limb.

In this study, we used ultrasonography to measure the PA of GM muscle in patients with spastic hemiplegia due to chronic cerebral injury, since physical examinations are not sensitive to detect unobvious changes of properties of muscle. Ultrasonography used to measure the PA of GM muscle in stroke patients have been reported before (Cho et al., 2018; Gao, Grant, Roth, & Zhang, 2009; Gao & Zhang, 2008), and the reliability and validity were tested to be excellent (Cho et al., 2018). Under passive condition, a decreased PA indicated higher fascicular tension and was associated with increased muscle tone (Gao et al., 2009). In this study, the PA of GM muscle in patients with chronic cerebral injury was significantly smaller on the affected lower limb, when compared with the PA on the unaffected lower limb before the surgery, which was coincident with previous studies (Cho et al., 2014; Gao et al., 2009). Higher muscle tone on the affected side may mainly contribute to a smaller PA of muscle, and muscle atrophy on the affected lower limb observed in these chronic cerebral injury patients was also one reason for a smaller PA (Gao et al., 2009). Before the surgery, higher muscle tone and muscle atrophy existed in the affected lower limb, and the results of measured PA of GM muscle were consistent with clinical manifestation in these spastic hemiplegia patients. Besides, the test results of plantar load distribution of the lower limbs showed patients' postoperative plantar load distribution of affected forefoot was significantly reduced compared to that of preoperative load with backfoot load increasing significantly. It can also reflect the changes of muscle properties, which made contribution to reducing the extend of spastic foot‐drop.

We have reported that transferring contralateral C7 nerve can improve the function and reduce the spasticity of affected arm before (Zheng et al., 2018). In patients with spastic hemiplegia due to chronic cerebral injury in this study, the postoperative PA of GM muscle on affected side changed to be significantly larger than preoperative condition. This outcome indicated that peripheral nerve surgery on affected upper limb can also have effects on the affected lower limb, which may be undetectable by routine physical examinations. However, the postoperative PA of GM muscle on unaffected side did not change obviously compared with preoperative condition, which means that peripheral nerve surgery on affected upper limb has fewer or no effects on the unaffected lower limb than that on the affected lower limb. The results indicated that there may exist a link between the upper and lower limbs, and this interlimb interaction may be selective and adjustable.

**Figure 4:**
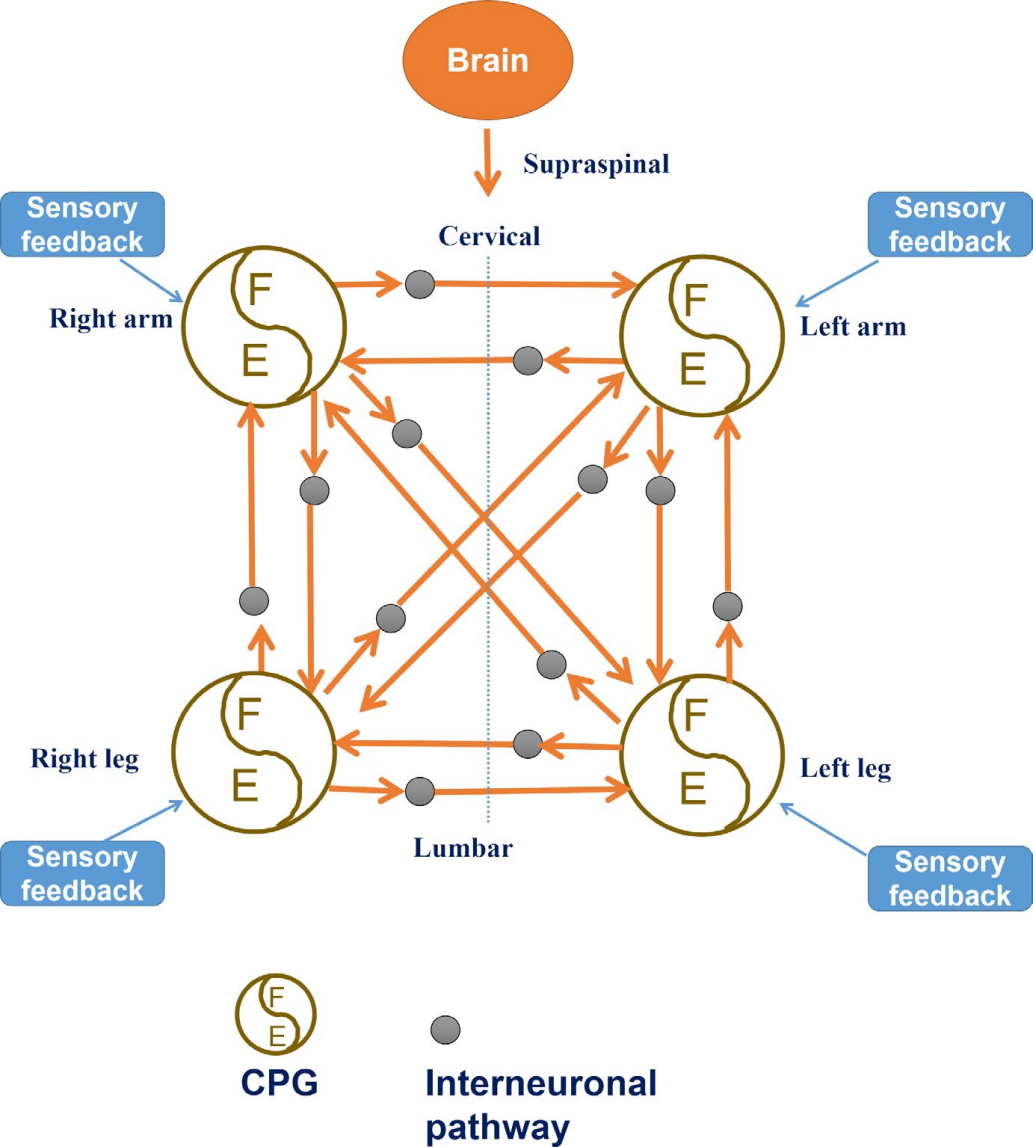
Schematic indicating the interlimb pathways and central pattern generator (CPG) with reference to literature (Kaupp et al., 2018; Klarner & Zehr, 2018; Minassian et al., 2017). Neuronal connections were represented with arrows and can be either excitatory or inhibitory. CPG for each limb can be affected and affect each other, and also receive sensory feedback input. E, extension; F, flexion

Hirsch et al. (2005) investigated the association between injecting botulinum toxin into spastic muscle of affected upper limb and changes in gait of lower limb in patients with hemiparesis after stroke. In all patients, stride time of the paralyzed leg was improved after BTX injection, which showed that upper extremity injection of botulinum toxin can affect the function of lower limb (Hirsch et al., 2005). The excitability of myotatic arc reflexes including quadriceps, biceps femoris, and soleus of lower limb can be affected by postural changes of upper limbs, which indicated acervicolumbar reflex interactions in human (Delwaide, Figiel, & Richelle, 1977). Fatiguing intermittent lower limb exercise also changed the excitability of muscle of unexcised upper limbs in healthy volunteers (Takahashi et al., 2011). The neural control mechanisms of interlimb coordination within the spinal cord mainly include intrinsic pathways in the spinal cord, various supraspinal pathways, and somato‐sensory feedback from the limbs (Frigon, 2017; Ramirez‐Jarquin & Tapia, 2018). The basic locomotor output may be controlled by the central pattern generators (CPGs) located in cervical and lumbar, and there are strong interactions between cervical and lumbar CPGs (Frigon, 2017; Kaupp et al., 2018; Klarner & Zehr, 2018; Minassian, Hofstoetter, Dzeladini, Guertin, & Ijspeert, 2017; Ramirez‐Jarquin & Tapia, 2018; Figure 4). However, the studies on neural control mechanisms of interlimb coordination were all conducted in animal models, which may have limitations as humans are very different in coordinating the limbs. Therefore, further new studies on interlimb interactions with new technologies are needed to be conducted in human. At present, interlimb coordination has not been paid enough attention in the clinics, especially in rehabilitation. Neurosurgeons and rehabilitation therapists often focus on the only affected upper limb or the affected lower limb. This study gained the importance of synergistic rehabilitation strategy of upper and lower limbs in patients with spastic hemiplegia due to central nerve injury.

Some limitations of this study should be noted. First, the number of cases is relatively small, so further clinical trials with more patients and rehabilitation alone as control group are needed, and longer period of following up is also necessary. Second, this study focused on changes of the PA with ultrasonography. Surface electromyogram and a three‐dimensional computerized motion analysis system are also needed to record corresponding changes of electrophysiology of muscles and gait in further study. Finally, the correlations analysis of changes in muscle parameters between upper and lower limbs should also be conducted in further study.

## 5 CONCLUSIONS

In conclusion, the findings in the present study indicate that contralateral C7 nerve transfer for improving the function of upper limb can also change muscle properties of lower limb in spastic hemiplegia patients. It revealed a link between the upper and lower limbs, which should be considered in rehabilitation physiotherapy for spastic hemiplegia patients due to chronic cerebral injury.

## CONFLICT OF INTEREST

None declared.

## Data Availability

The data that support the findings of this study are available from the corresponding author upon reasonable request.
